# Supplementary material for: Activated protein C protects from GvHD via PAR2/PAR3 signalling in regulatory T-cells
Source: Nat Commun. 2017 Aug 21;8:311. doi: 10.1038/s41467-017-00169-4 (PMC5566392; doi:10.1038/s41467-017-00169-4)
Supplement: Supplementary file 1 — Supplementary Information [file 41467_2017_169_MOESM1_ESM.pdf]

File name: Supplementary Information
Description: Supplementary figures and supplementary table.

File name: Peer review file
Description:

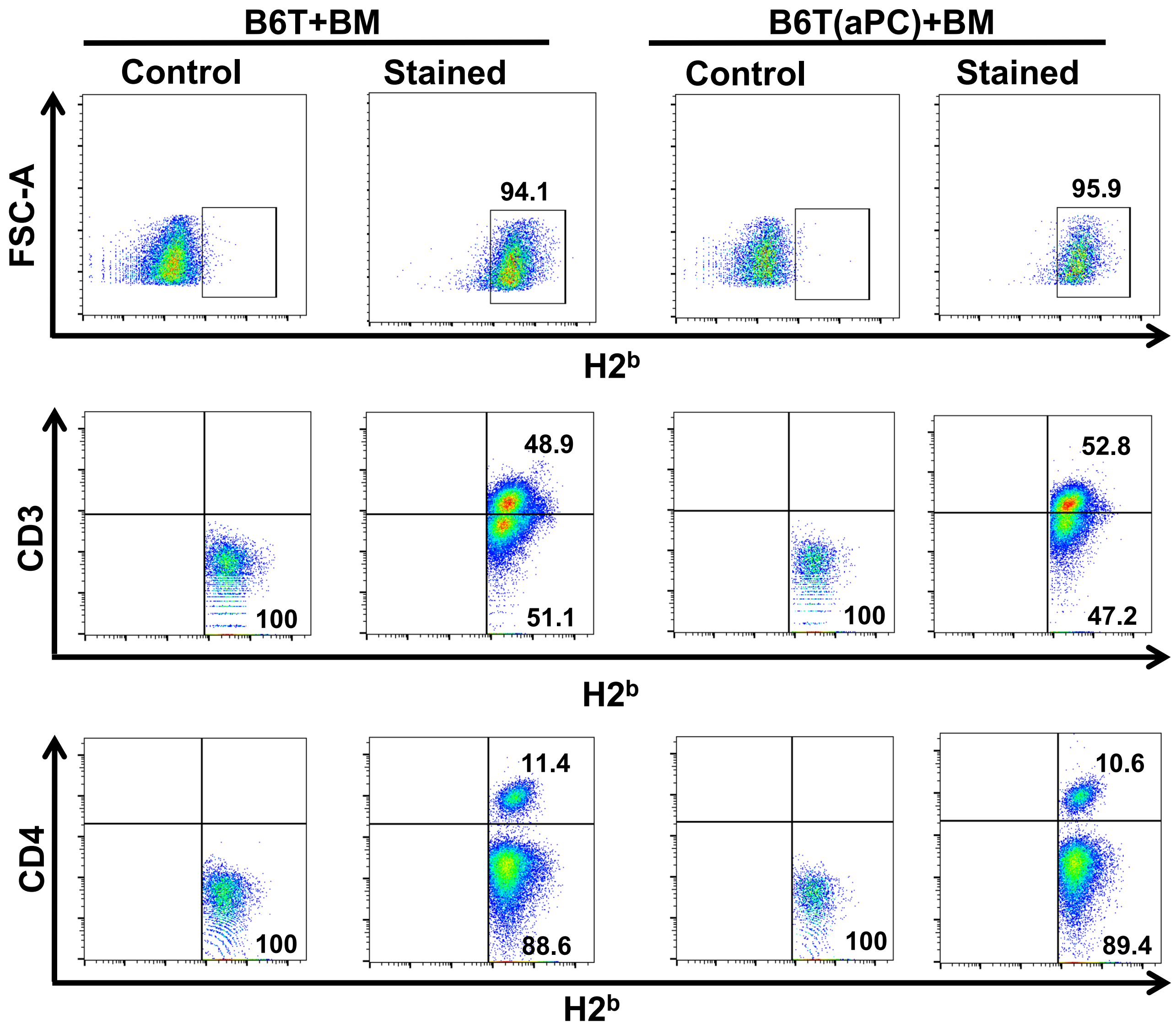

Supplementary Fig. 1: Engraftment of donor cells ($H2^{b+}$) and donor $CD3^+$ and $CD4^+$ T-cells is not altered by aPC-preincubation of T-cells

Recipient BALB/c mice were lethally irradiated (11Gy) and transplanted with 5×10^6 bone marrow and 0.5×10^6 T-cells without (B6T+BM) or with (B6T(aPC)+BM) aPC-preincubation (20nM, 1h, 37°C) from donor C57BL/6 wt mice. Recipient mice were sacrificed on day 3 post transplantation, splenic cells were harvested and stained for $H2^b$, CD3, or CD4 and analysed by flow cytometry. Control: unstained (top) or stained for $H2^b$ only (middle, bottom). Representative FACS images are shown.

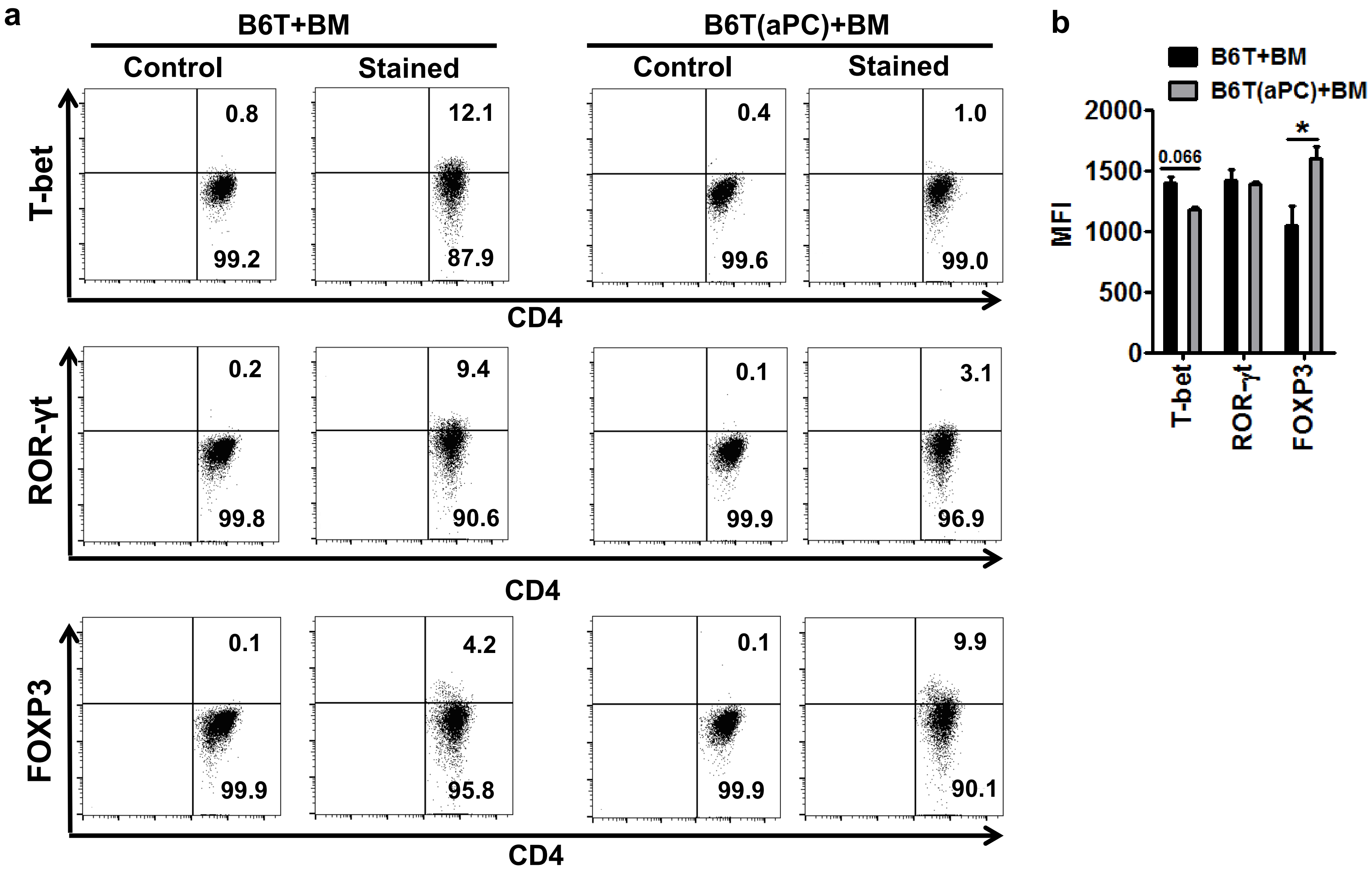

Supplementary Fig. 2 : Exemplary FACS-scans and MFI corresponding to Fig. 3e

Recipient BALB/c mice were lethally irradiated (11Gy) and transplanted with 5×10^6 bone marrow and 0.5×10^6 T-cells without (B6T+BM) or with (B6T(aPC)+BM) aPC-preincubation (20nM, 1h, 37°C) from donor C57BL/6 wt mice. Recipient mice were sacrificed 2 weeks post transplantation, splenic T-cells were harvested and stained for $H2^b$, CD4, T-bet, ROR-γt, or FOXP3 and analysed by flow cytometry. For T-bet, ROR-γt, and FOXP3 cells were gated on $H2^{b+}CD4^+$ cells. Control: stained for $H2^b$ and CD4. Representative FACS images (a) and Mean Fluorescence Intensity (b, MFI, Mean value ± SEM; N=5 each group) are shown; *P<0.05 (b: t-test).

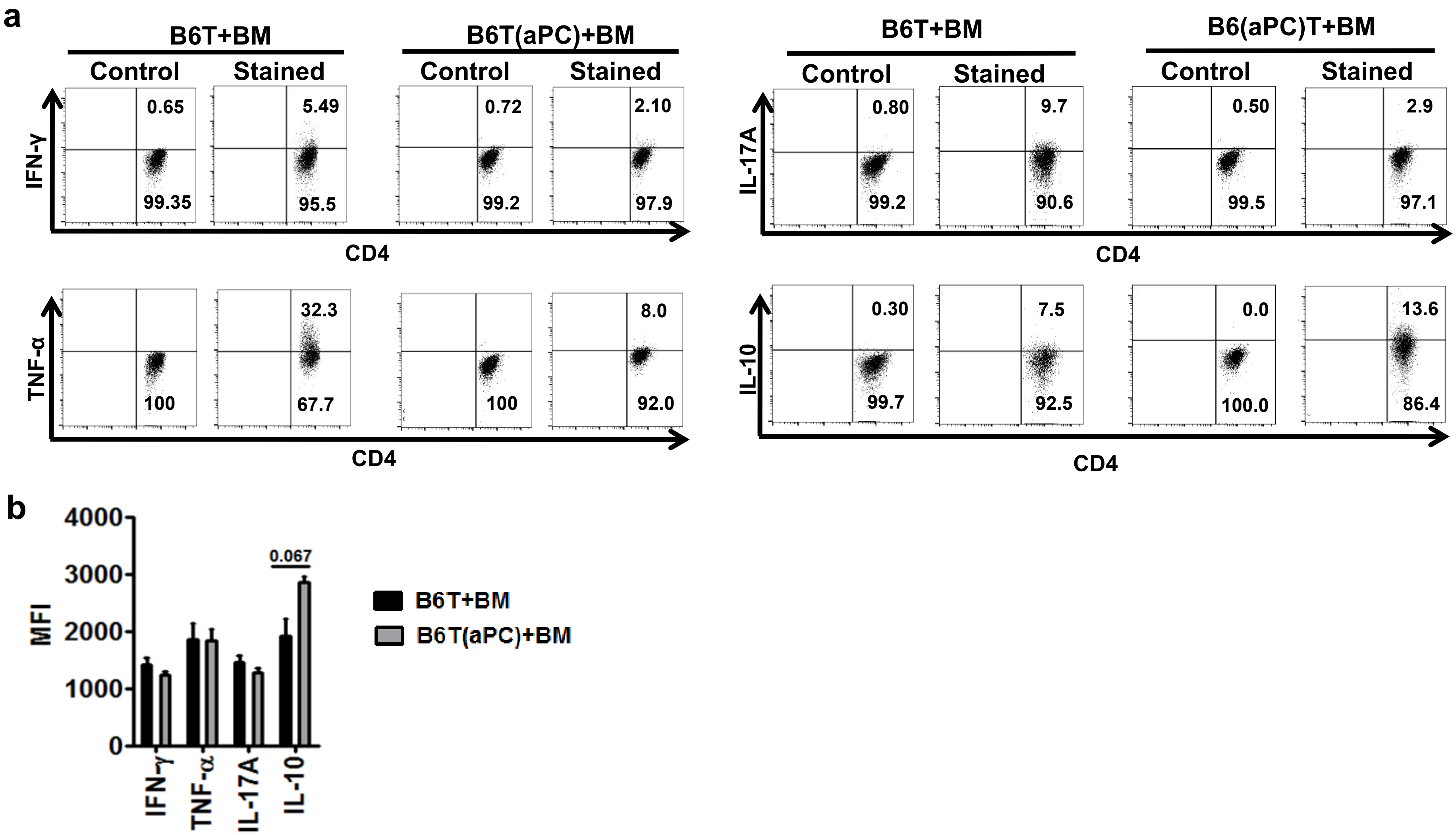

Supplementary Fig. 3: Exemplary FACS-scans and MFI corresponding to Fig. 3f

Recipient BALB/c mice were lethally irradiated (11Gy) and transplanted with 5×10^6 bone marrow and 0.5×10^6 T-cells without (B6T+BM) or with (B6T(aPC)+BM) aPC-preincubation (20nM, 1h, 37°C) from donor C57BL/6 wt mice. Recipient mice were sacrificed 2 weeks post transplantation, splenic T-cells were harvested and stained for $H2^b$, CD4, IFN-γ, TNF-α, IL-17A, or IL-10 and analysed by flow cytometry. For IFN-γ, TNF-α, IL-17A, and IL-10 cells were gated on $H2^{b+}CD4^+$ cells. Control: stained for $H2^b$ and CD4. Representative FACS images (a) and Mean Fluorescence Intensity (b, MFI, Mean value ± SEM; N=5 each group) are shown; (b: t-test).

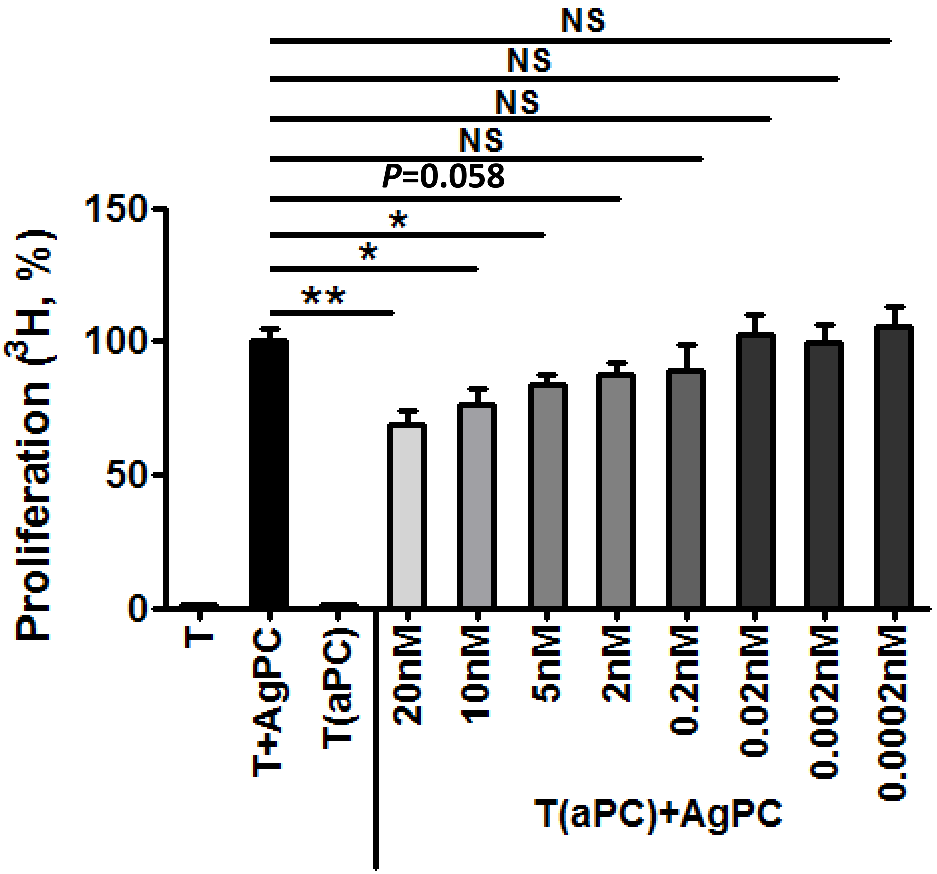

Supplementary Figure 4: Dose-response of aPC in the MLR

Following preincubation of human pan-T-cells with various concentrations of aPC (concentration as indicated, preincubation for 1h in AIM V medium) T-cells were co-cultured with irradiated human allogenic antigen-presenting cells for 96h. Allogenic T-cell reactivity was measured by thymidine incorporation during the final 16h. Mean value ± SEM of at least 3 independent experiments, each containing cells from three different donors; *$P<0.05$, **$P<0.01$, NS: not significant (t-test).

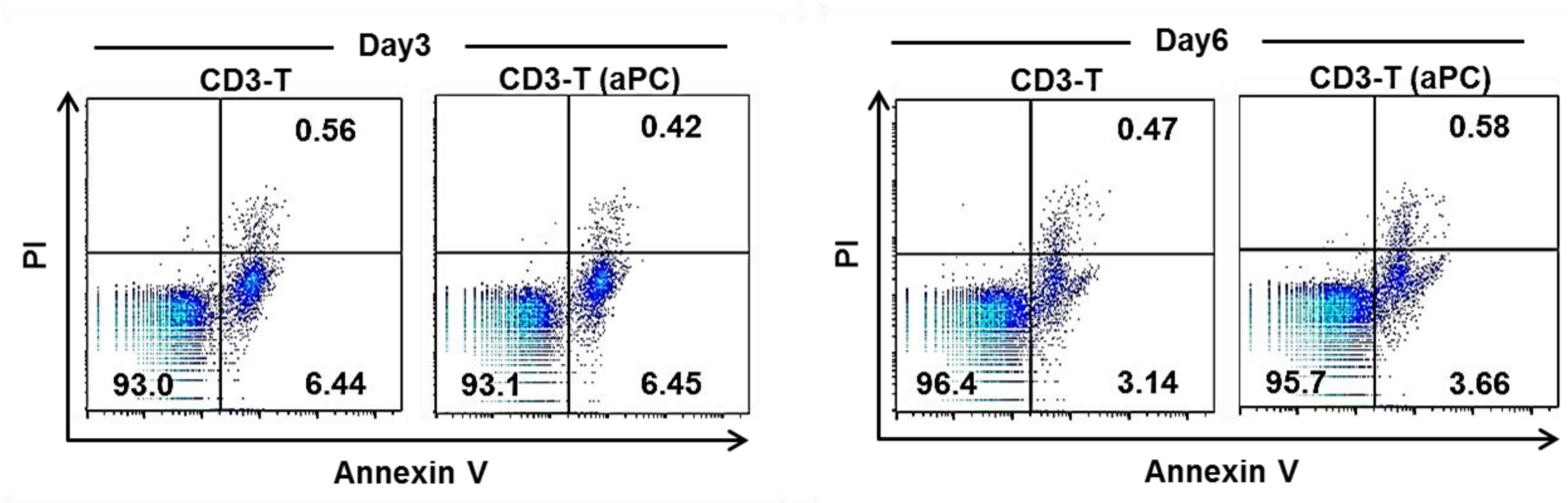

Supplementary Fig. 5: aPC has no impact on apoptosis in allogenic stimulated human T-cells
Human T-cells were stimulated with allogenic AgPC for 3 or 6 days and apoptosis was determined by annexin V and PI staining in CD3 T-cells. Representative FACS images of three independent repeat experiments are shown.

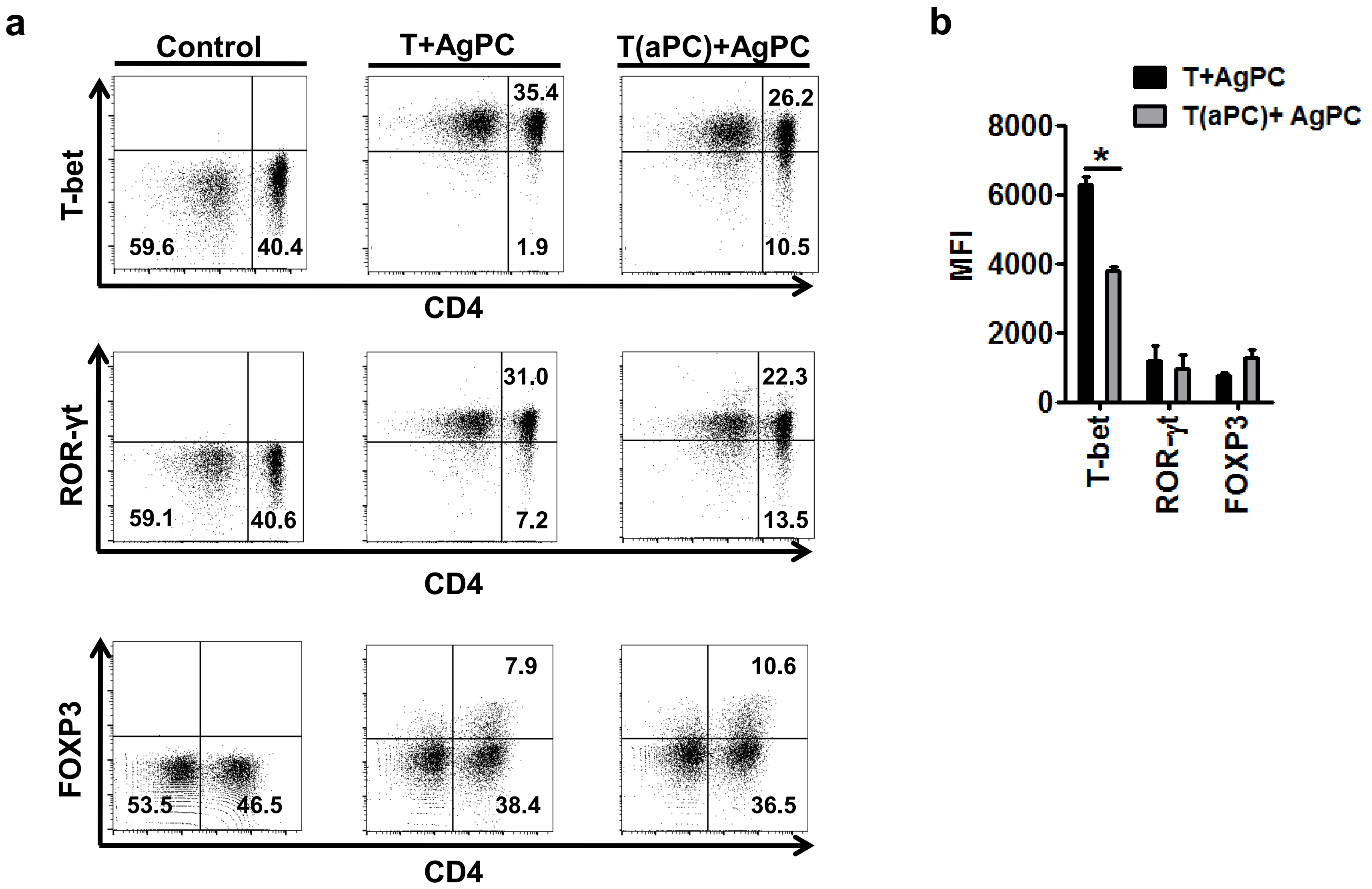

Supplementary Fig. 6: Exemplary FACS-scans corresponding to Fig. 4d
aPC-preincubation of human pan T-cells reduces the frequency of $CD4^{+}$T-bet^{+} and $CD4^{+}$ROR-γt^{+} T-cells, while increasing the frequency of $CD4^{+}FOXP3^{+}$ regulatory T-cells, as measured by flow cytometry after 48h of MLR. For T-bet, ROR-γt, and FOXP3 cells were gated on lymphocytes. Control: stained for CD4 only. Representative FACS images (a) and Mean Fluorescence Intensity (b, MFI, Mean value ± SEM; N=5 each group) are shown; *P<0.05 (b: t-test).

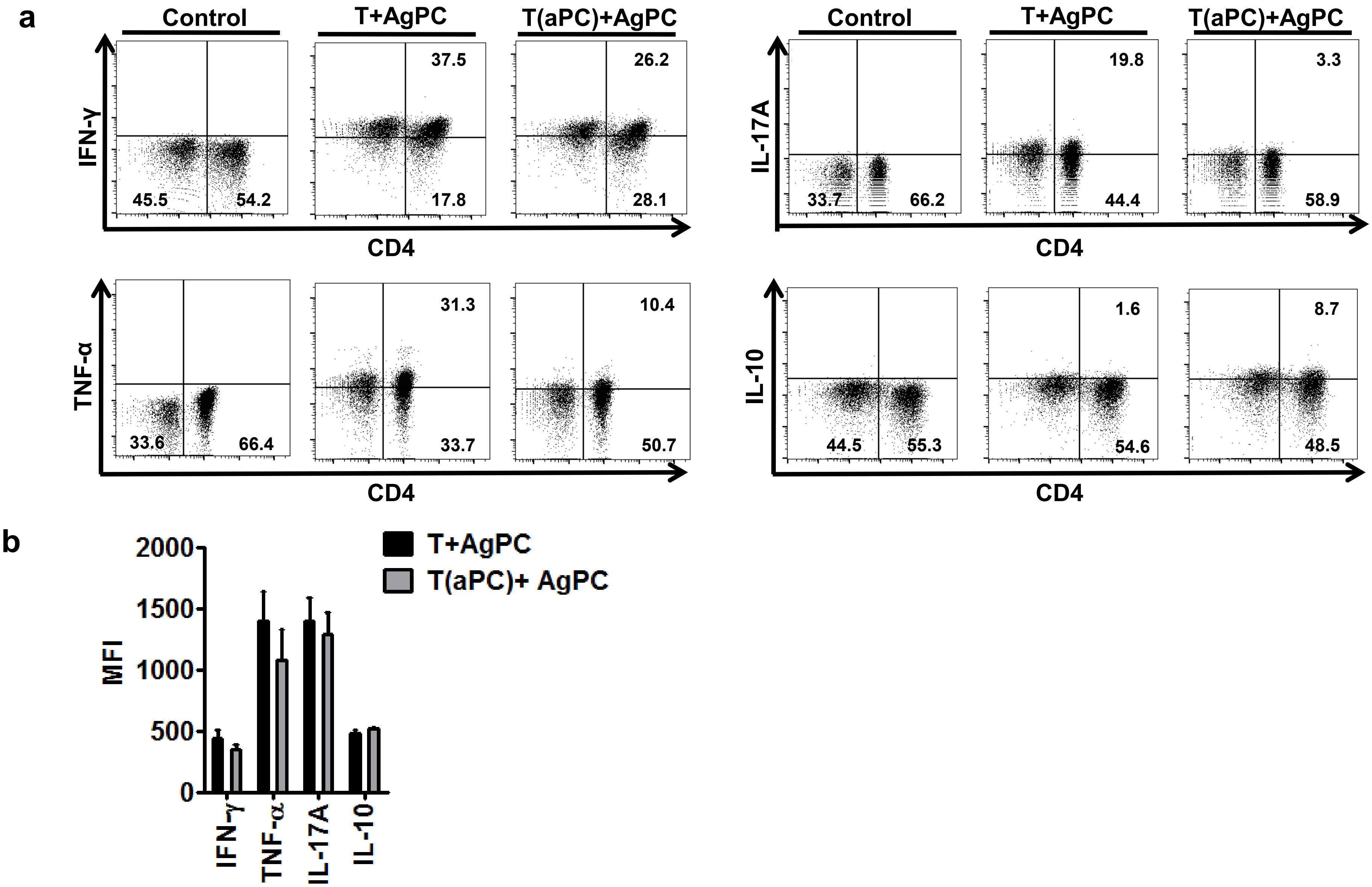

Supplementary Fig. 7: Exemplary FACS corresponding to Fig. 4e

aPC-preincubation of human pan T-cells reduces expression of the pro-inflammatory cytokines IFN-γ, TNF-α, and IL-17A, while that of IL-10 is increased as measured by flow cytometry after 48h of MLR. For IFN-γ, TNF-α, IL-17A, or IL-10, cells were gated on lymphocytes. Control: stained for CD4 only. Representative FACS images (a) and Mean Fluorescence Intensity (b, MFI, Mean value ± SEM; N=5 each group) are shown.

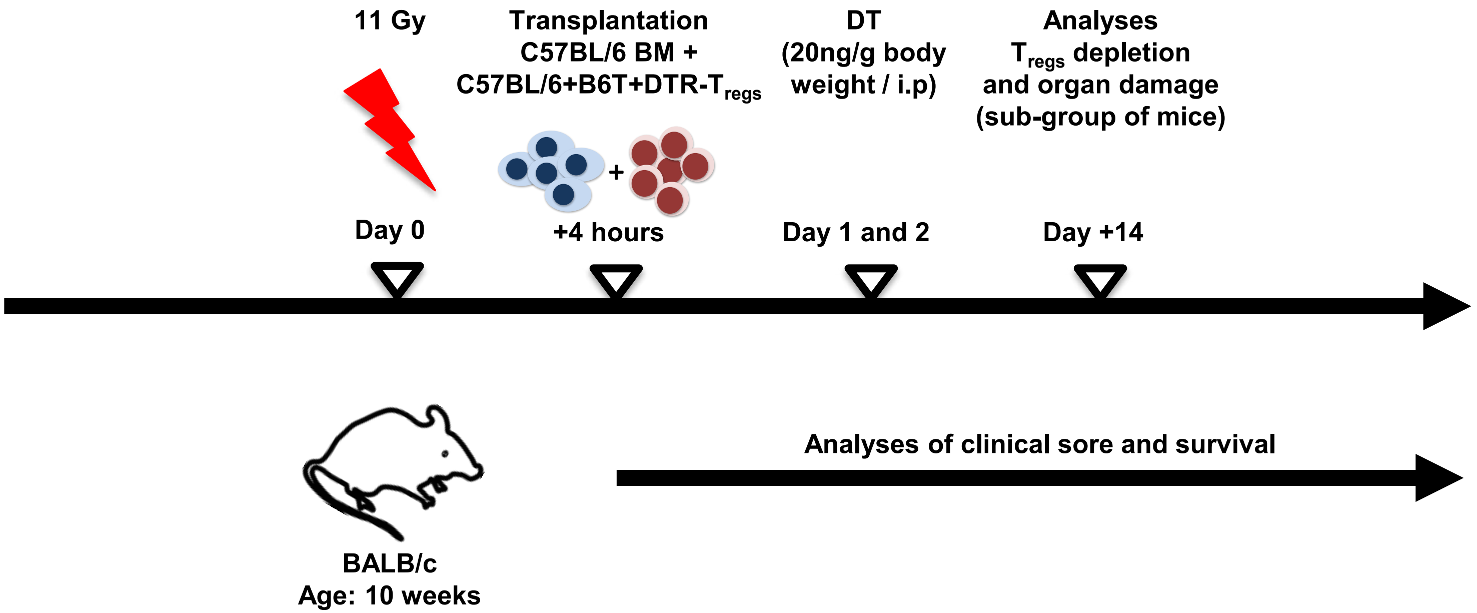

Supplementary Fig. 8: Scheme presenting experimental approach of transplantation of DTR-T_{reg} in mice.
Experimental approach for depletion of T_{regs} in mice transplanted with DEREG derived T_{regs}. BALB/c mice, age 10 weeks, were irradiated with a single dose (11 Gy). Post-irradiation (after 4h) mice were transplanted with C57BL/6 derived bone marrow cells (5 x 10^6), C57BL/6 derived T-cells (0.4 x 10^6) and T_{regs} (0.1 x 10^6) obtained from DEREG mice expressing the diphtheria toxin receptor (DTR) and eGFP under the control of the FOXP3 promoter. T_{regs} were preincubated with aPC (20 nM, in AIM V medium) or left untreated (only AIM V medium) for 1h. On day 1 and 2 mice were injected with diphtheria toxin (DT, 20ng/g body weight /intraperitoneal injection (i.p)). Depletion of T_{regs} was ascertained on day 14 by isolating splenocytes and determining the frequency of GFP expressing T_{regs} cells in a subgroup of mice. Other mice were continuously monitored and a clinical score to quantitate GvHD severity was generated. See material and methods section for further details.

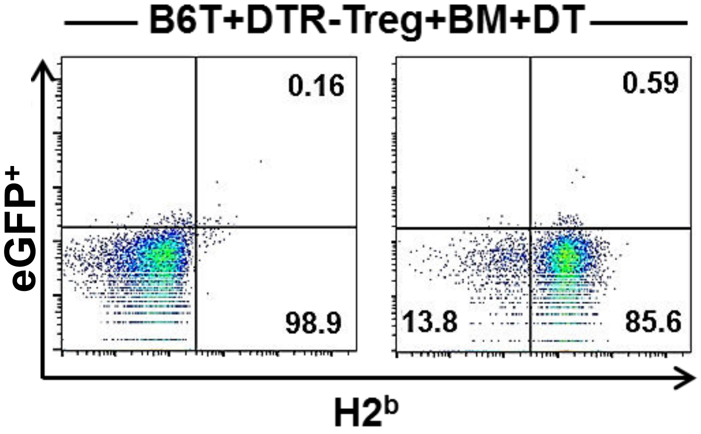

Supplementary Fig. 9: Depletion of donor GFP$^+$DTR$^+$-T$_{regs}$

Recipient mice were transplanted with C57BL/6 derived bone marrow cells (5 x 10^6), C57BL/6 derived T-cells (0.4 x 10^6) and T$_{regs}$ (0.1 x 10^6) obtained from DEREG mice on day 0 and injected with DT (20ng/g body weight, i.p.) on day 1 and 2 (B6T+DTR-Treg+BM+DT). Splenic donor cells (H2^{b+}) were analysed for GFP$^+$ DTR-Treg in transplanted mice by FACS on day 14. Exemplary FACS images shown (left: H2^b negative control, right: H2^{b+}eGFP$^+$ T$_{regs}$).

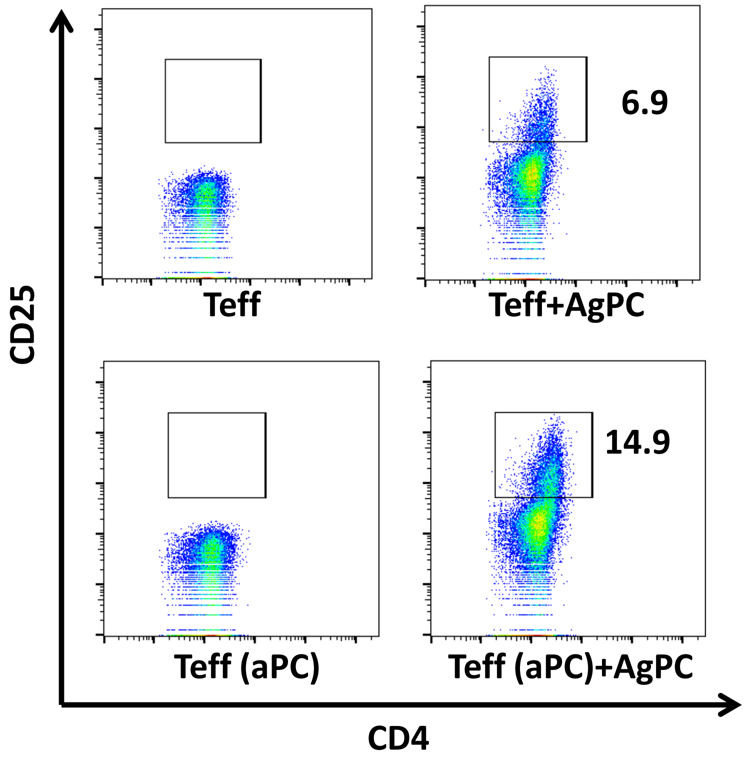

Supplementary Fig. 10: Exemplary FACS corresponding to Fig 5e
Preincubation of human effector T-cells ($CD4^{+}CD25^{-}$) with aPC induces regulatory cells ($CD4^{+}CD25^{+}$). Effector T-cells without (T_{eff}) and with (T_{eff}(aPC)) aPC-preincubation were stimulated with AgPC for 96h. Cells were measured by FACS and analysed by FlowJo software. Representative FACS images are shown.

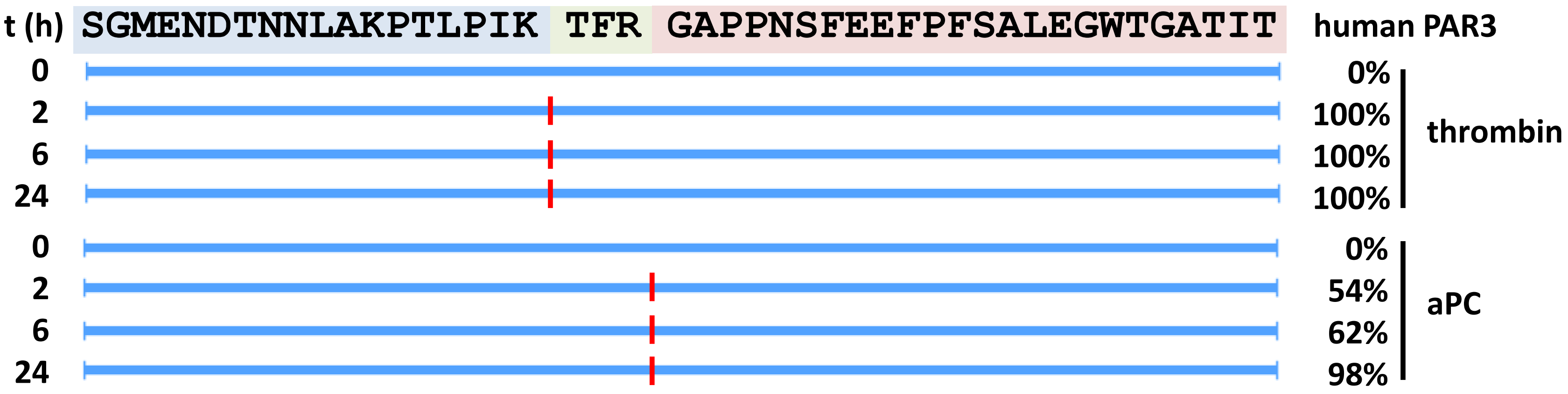

Supplementary Fig. 11: Summary of human PAR3-N-terminal peptide cleavage analyses. A peptide derived from the N-terminal end of human PAR3 (sequence shown at the top) was incubated with human thrombin (10 nM) or human aPC (500 nM). Proteolysis was determined at indicated time-points (left, t(h)). Cleavage sites are shown in red lines and estimated cleavage efficiency is shown in percentage (right).

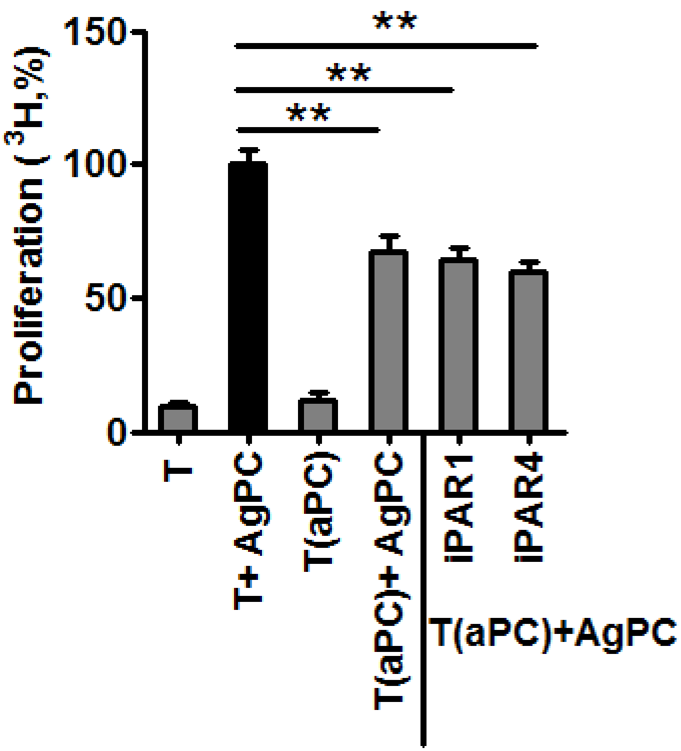

Supplementary Fig. 12: Inhibitory peptides against PAR1 and PAR4 failed to abolish aPC's effect in the MLR
Preincubation of human peripheral blood T-cells (T) with inhibitory peptides against PAR1 (iPAR1) or PAR4 (iPAR4) prior to aPC-preincubation and MLR does not abrogates aPC's inhibitory effect in regard to T-cell reactivity. Mean value ± SEM); **P<0.01 (t- test).

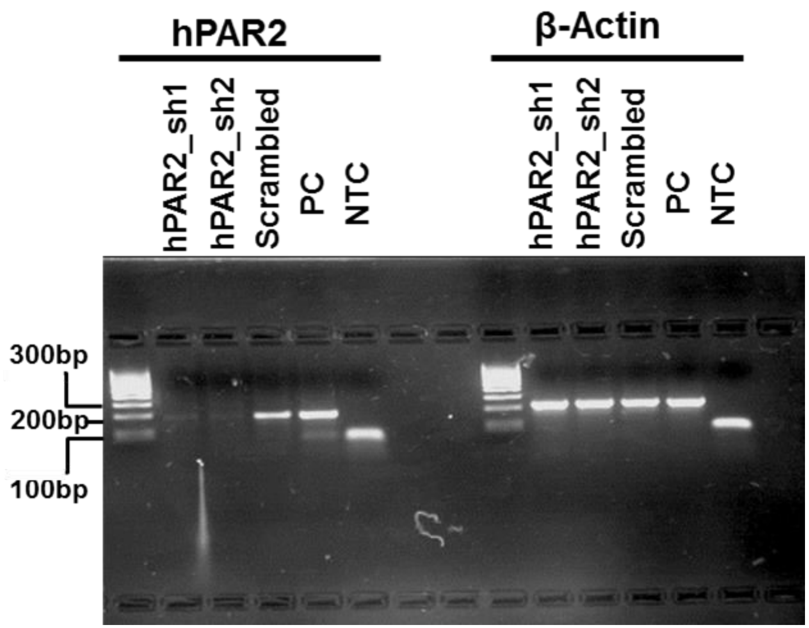

Supplementary Fig. 13: Knock down of PAR2 in primary human T-cells. Primary human T-cells were transfected with lentiviral particles mediating expression of two disjunct shRNA (hPAR2_sh1, hPAR2_sh2) against human PAR2 (178bp) or a control shRNA (scrambled). Both shRNAs efficiently suppressed expression of PAR2, while the control shRNA had no effect. Beta actin (β-Actin-227bp) was used as loading control. Exemplary image of an RT-PCR analyses conducted on day 2 after transfection. SM: size marker, PC: positive control (non-transduced human pan-T-cells; NTC: negative control (no reverse transcriptase).

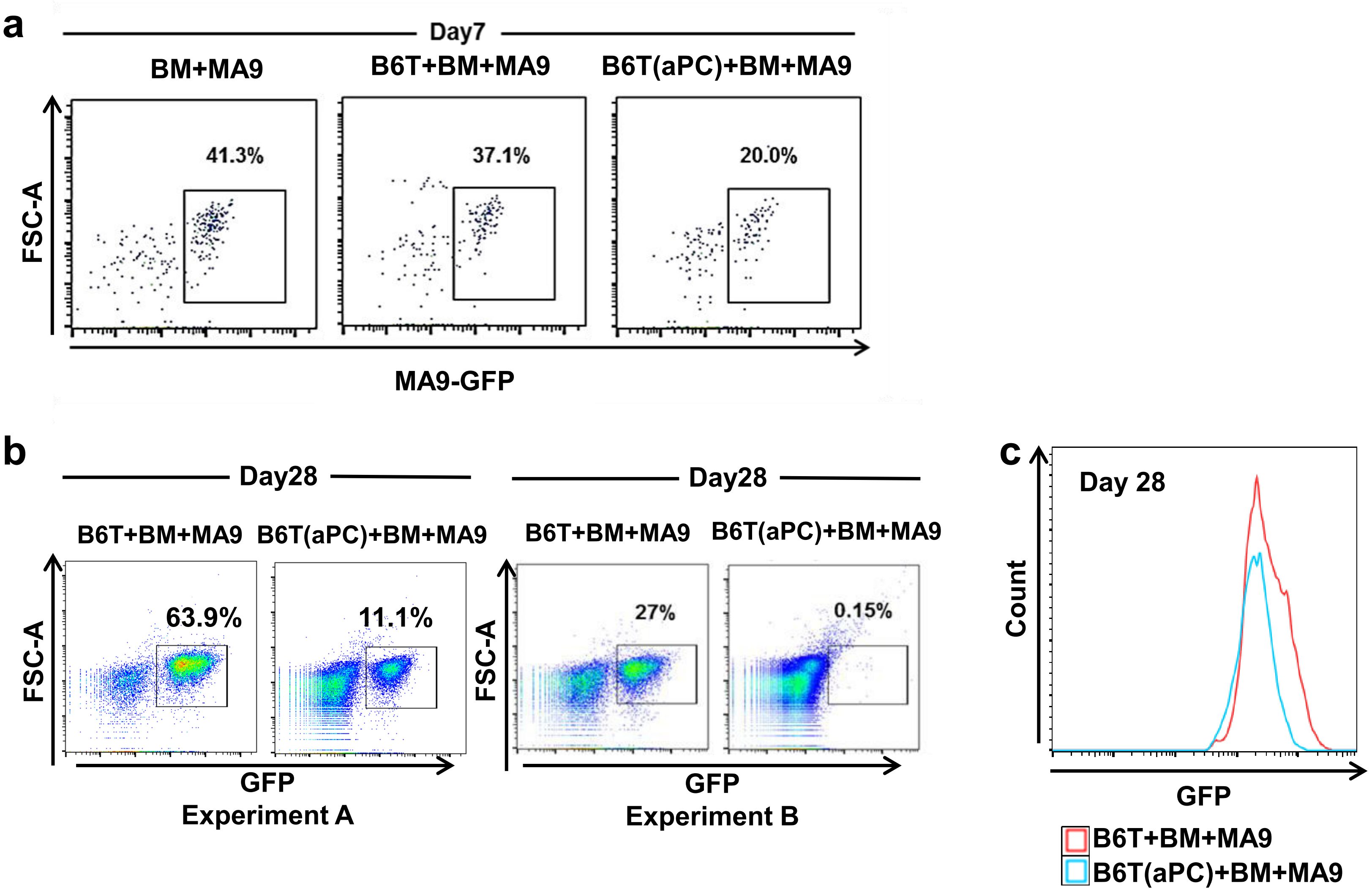

Supplementary Fig. 14: Exemplary FACS-scans corresponding to Fig. 10b,c
Lethally irradiated (11Gy) recipient BALB/c mice were transplanted with 5×10^6 C57BL/6 derived bone marrow cells, 5000 GFP+MA9 leukemic cells, and 0.5×10^6 C57BL/6 T-cells without (B6T+BM+MA9) or with (B6T(aPC)+BM+MA9) aPC-preincubation. In mice transplanted with aPC preincubated T-cells leukemic load (determined by flow cytometry on day 7 (a) and day 28 (b,c) after transplantation in peripheral blood samples) is markedly decreased. Representative FACS images (a,b) and histogram (c) are shown.

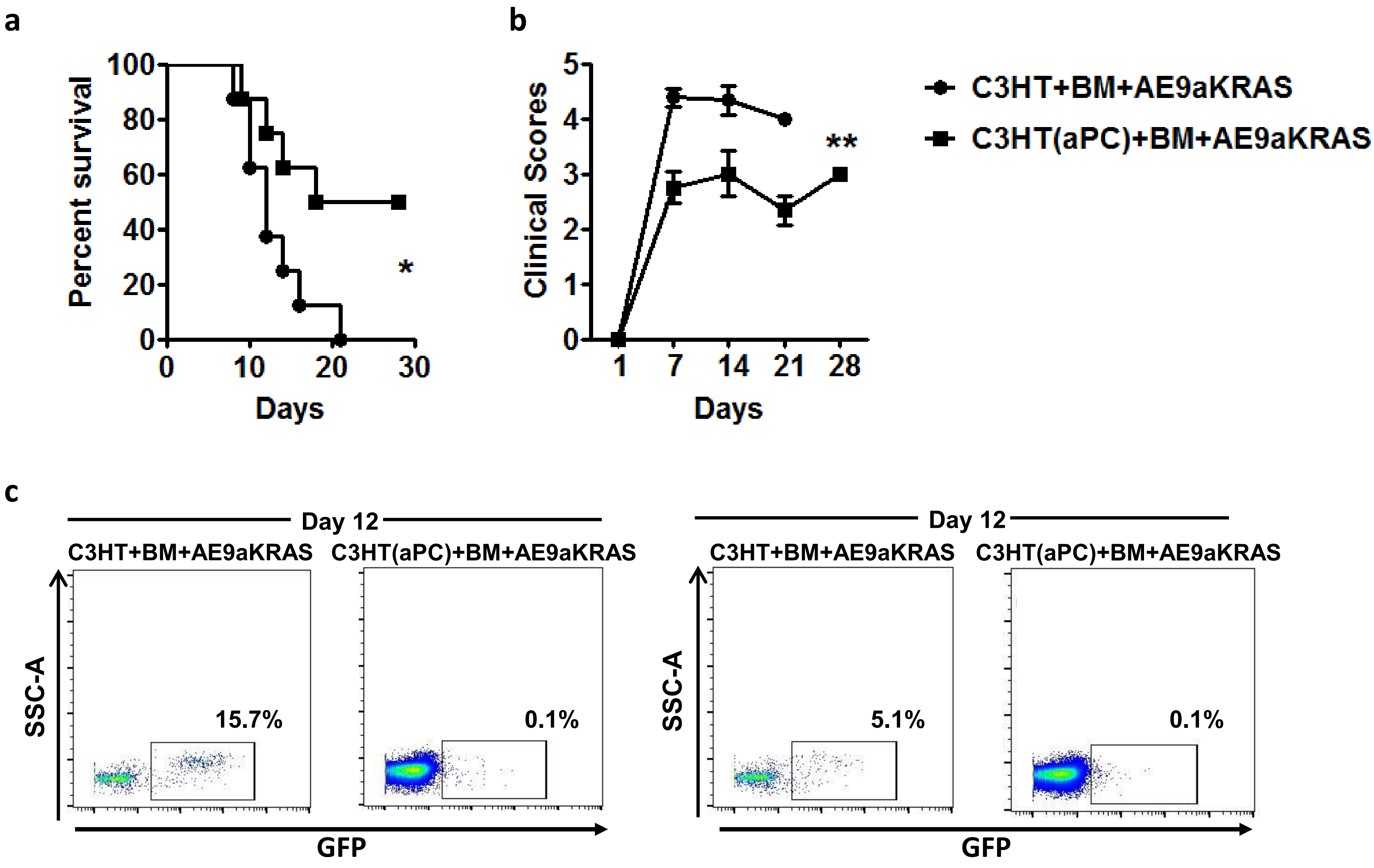

Supplementary Fig. 15: aPC preincubation of T-cells does not impair GvL effect

Lethally irradiated (13Gy) recipient C57BL/6 mice were transplanted with 5×10^6 C3H derived bone marrow cells, 2×10^4 GFP^+AE9aKRAS leukemic cells, and 1×10^6 C3H T-cells without (C3HT+BM+AE9aKRAS) or with (C3H(aPC)+BM+AE9aKRAS) aPC-preincubation. Survival curve (a) and clinical scores (b) are shown. In mice transplanted with aPC preincubated T-cells leukemic load (determined by flow cytometry on day 12 (c) after transplantation in peripheral blood samples) is markedly decreased. Representative FACS images (c) are shown.. Mean value ± SEM; N=8 each group) are shown; *P<0.05, **P<0.01 (log-rank test).

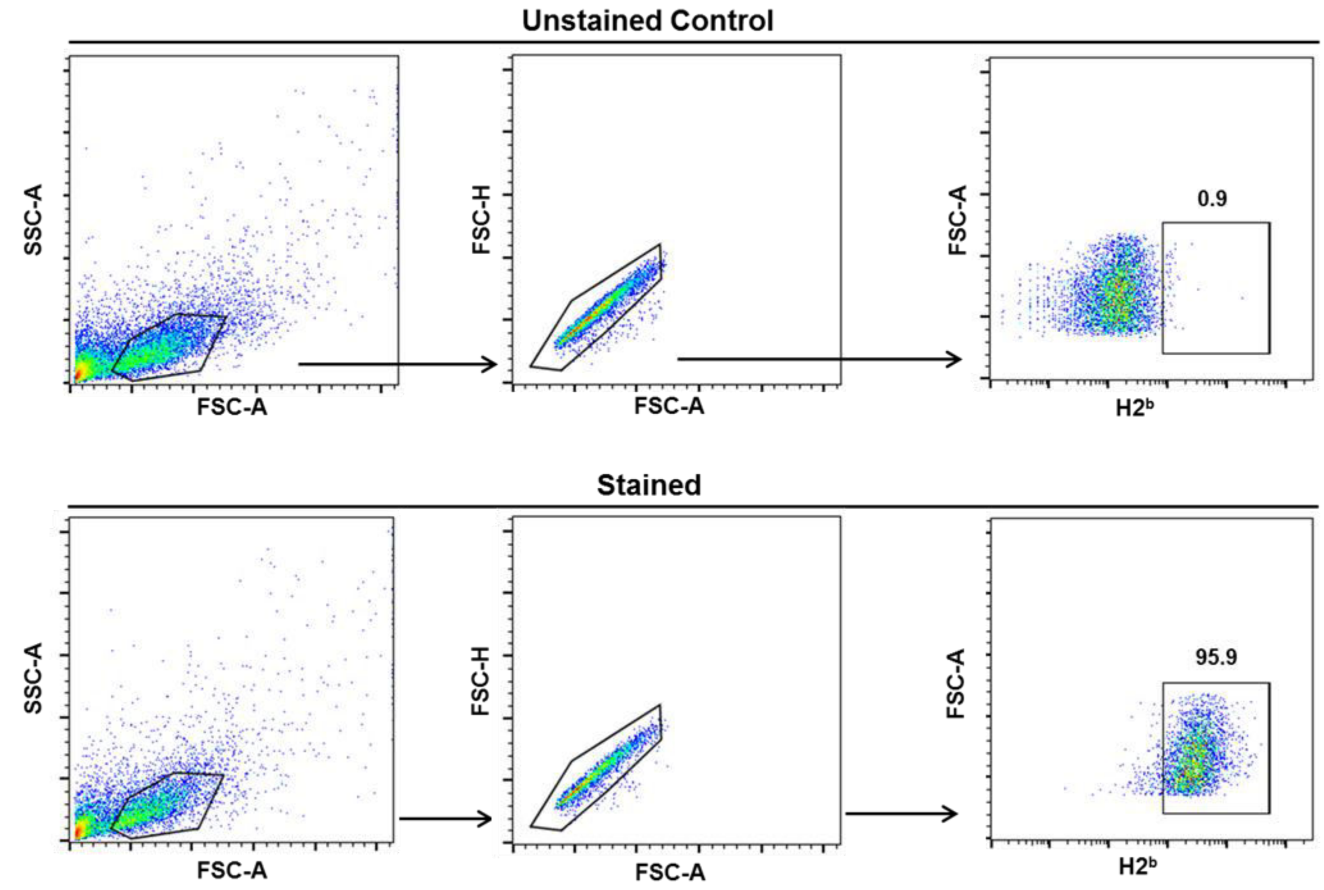

Supplementary Fig. 16: Representative gating strategy for unstained control and stained samples corresponding to Supplementary Figure 1 (part 1).

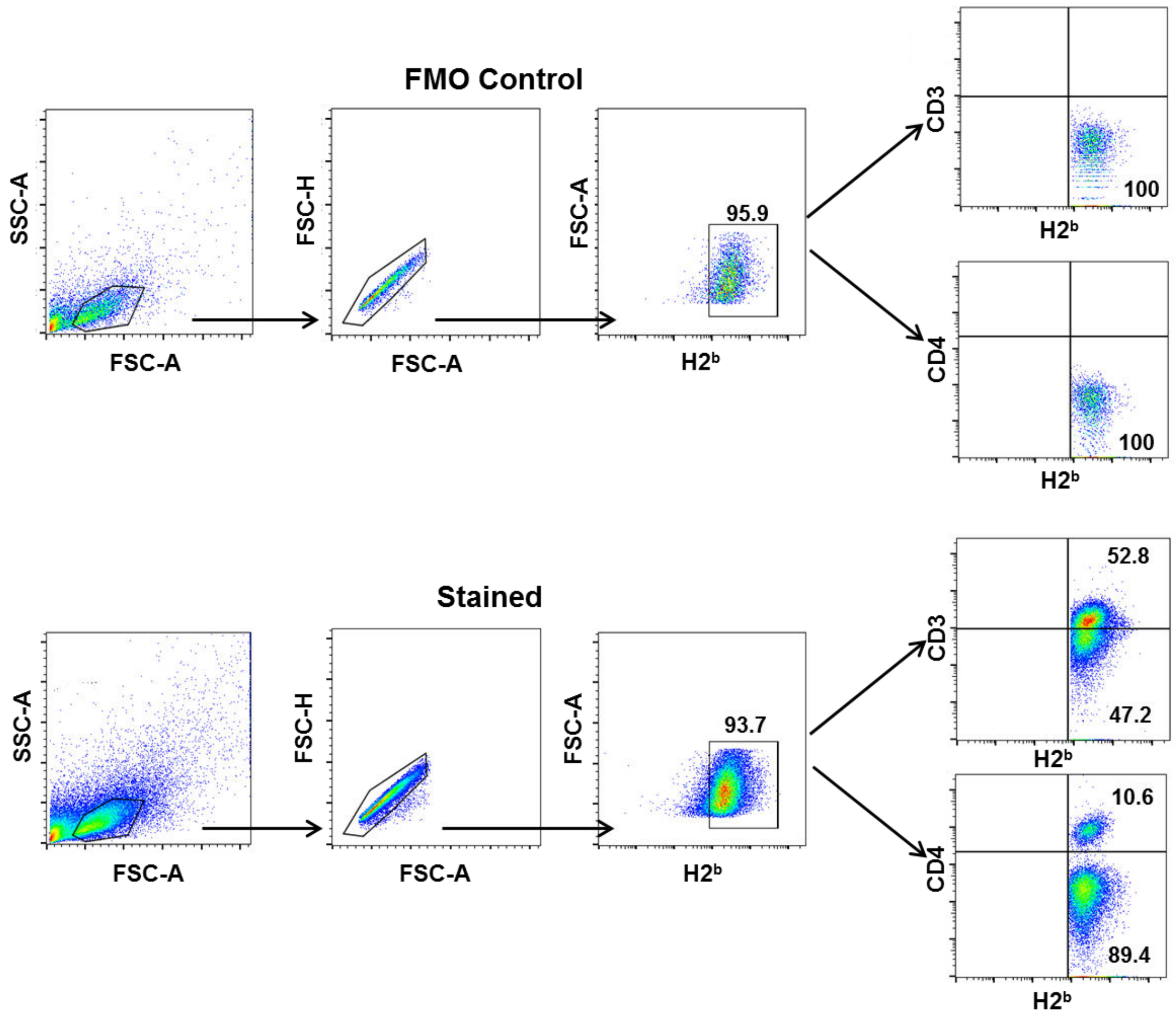

Supplementary Fig. 17: Representative gating strategy for FMO control and stained samples corresponding to Supplementary Figure 1 (part 2).

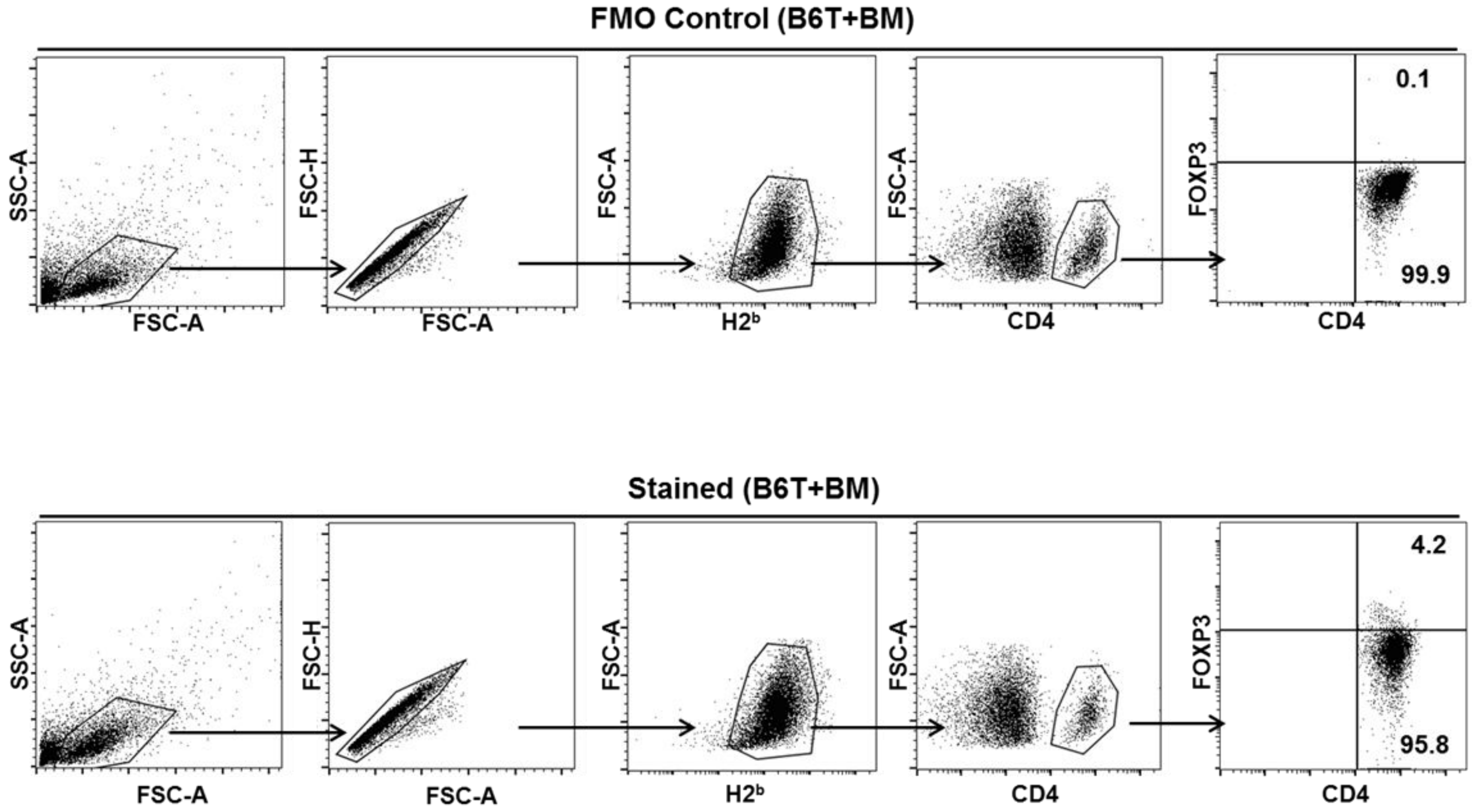

Supplementary Fig. 18: Representative gating strategy for FMO control and stained samples corresponding to Supplementary Figure 2.

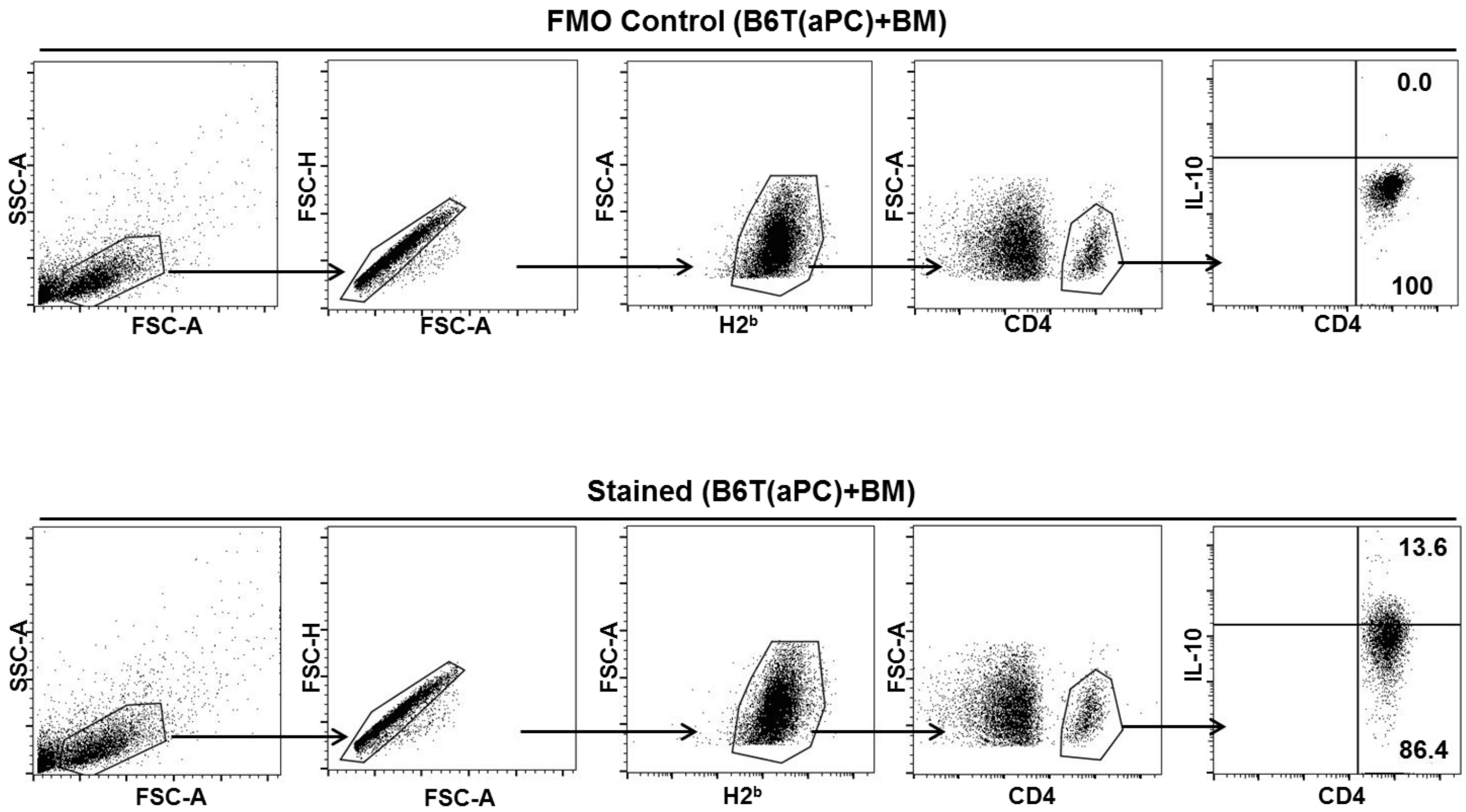

Supplementary Fig. 19: Representative gating strategy for FMO control and stained samples corresponding to Supplementary Figure 3.

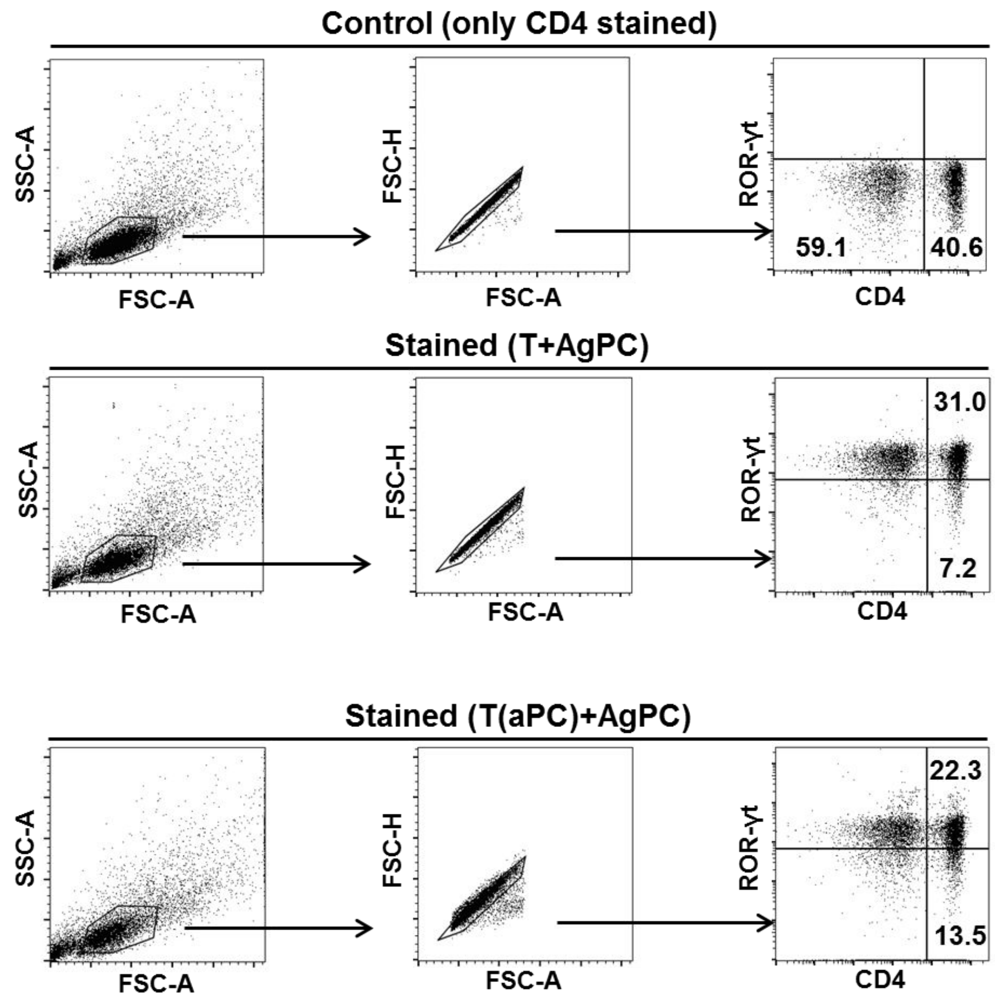

Supplementary Fig. 20: Representative gating strategy for control and stained samples corresponding to Supplementary Figure 6.

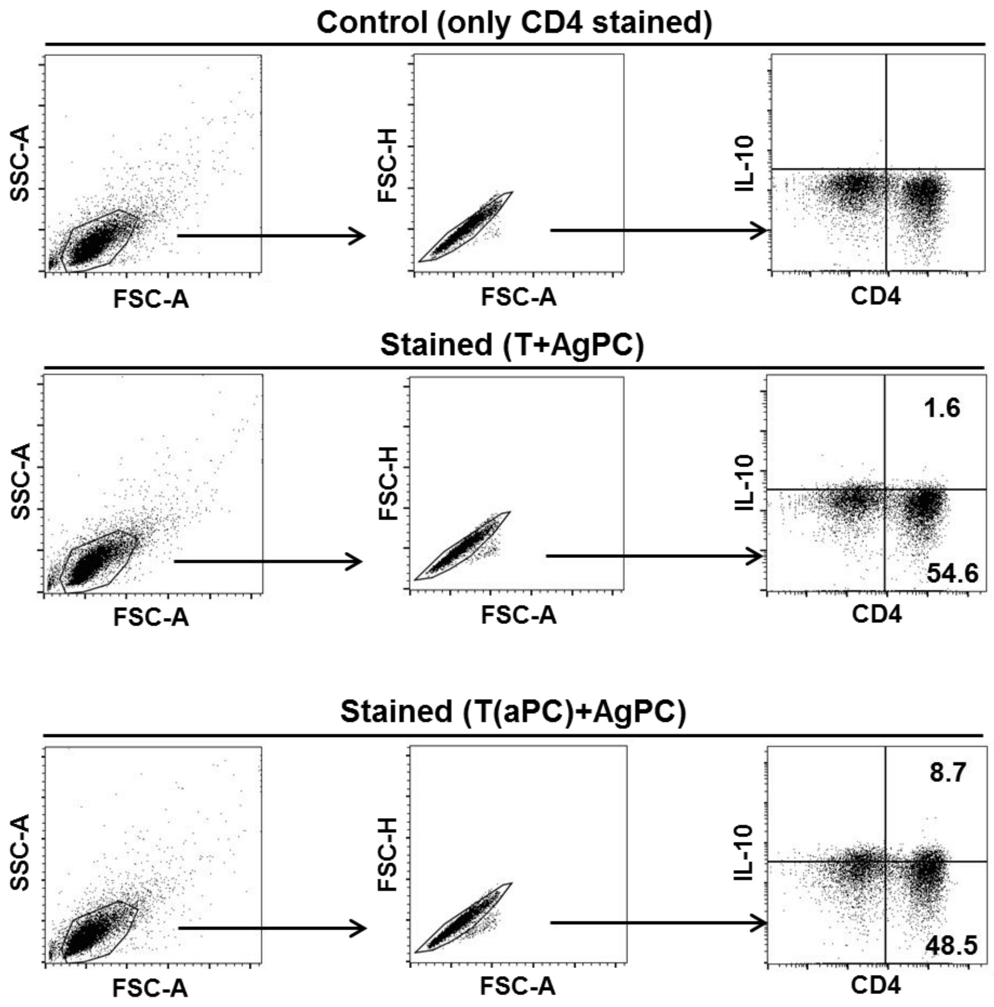

Supplementary Fig. 21: Representative gating strategy for control and stained samples corresponding to Supplementary Figure 7.

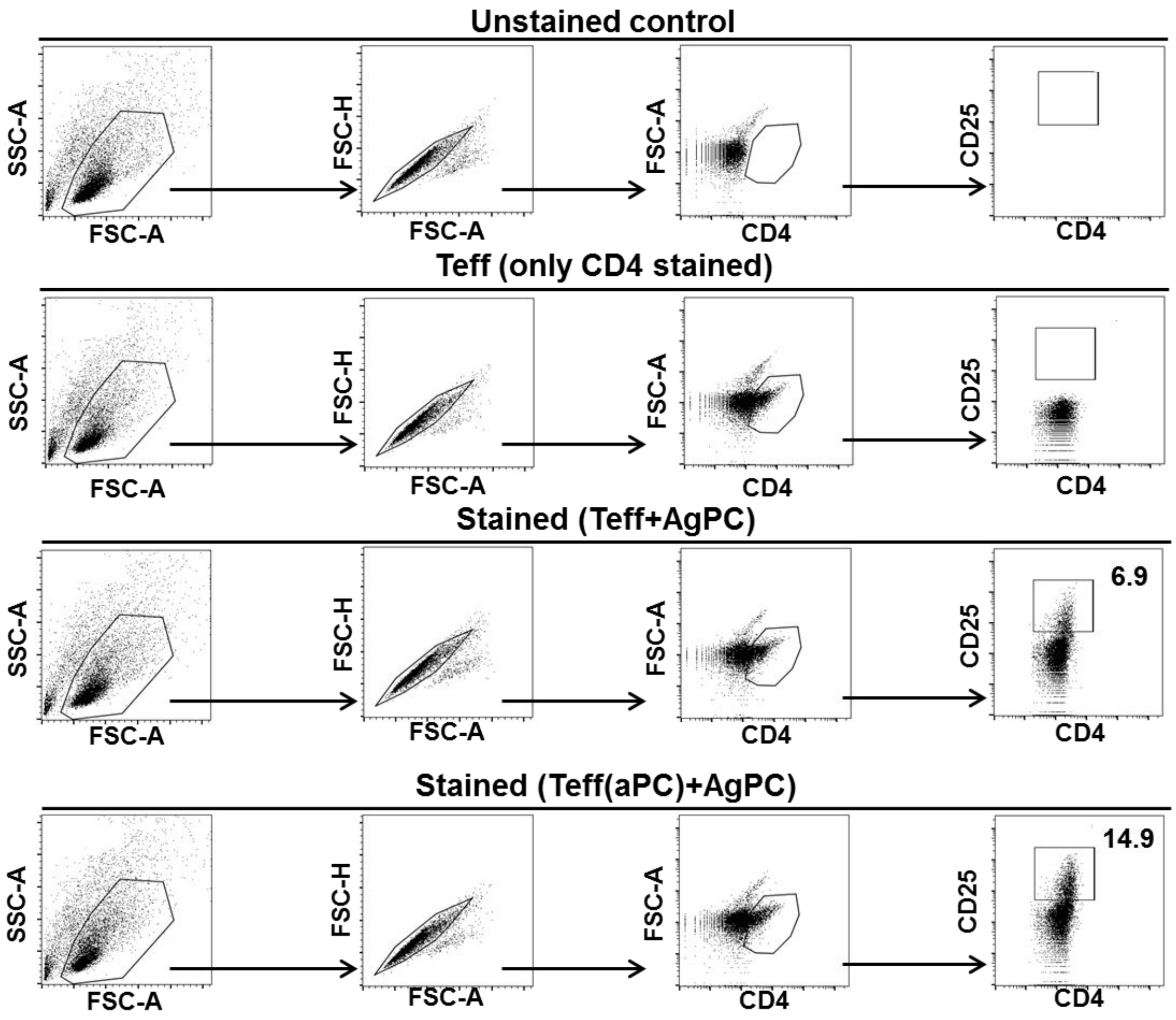

Supplementary Fig. 22: Representative gating strategy for control and stained samples corresponding to Supplementary Figure 10.

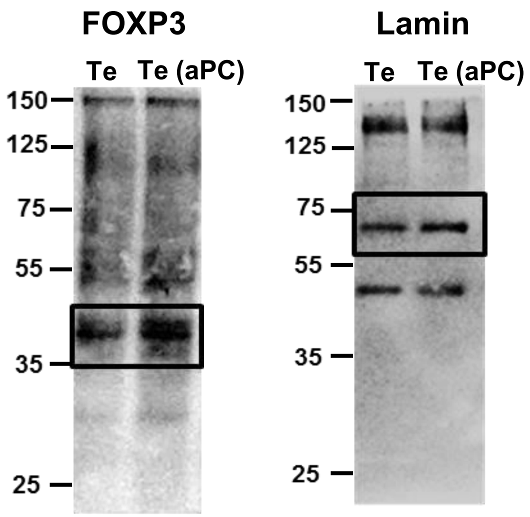

Supplementary Fig. 23: Full western blot images corresponding to Fig. 5f.

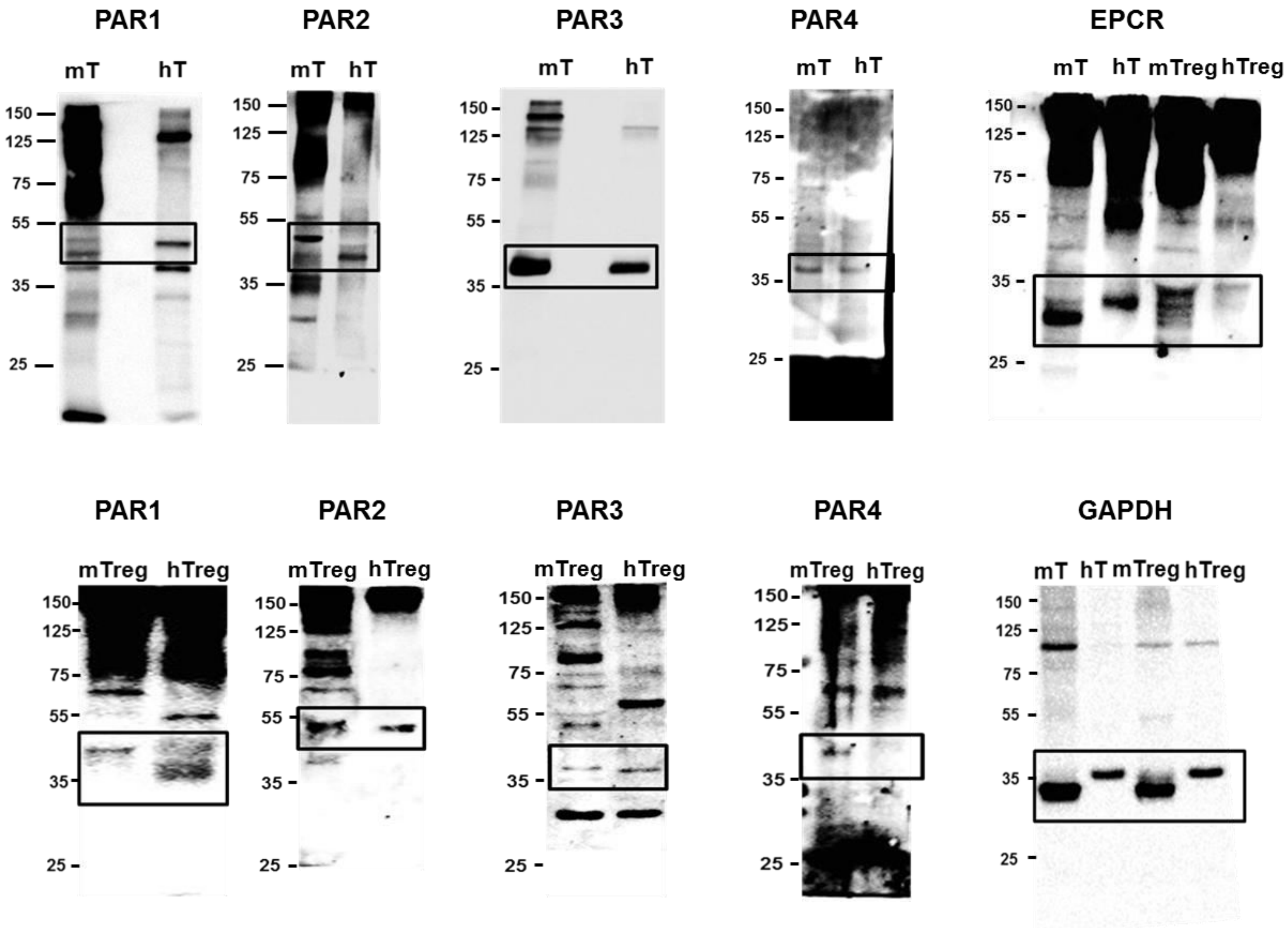

Supplementary Fig. 24: Full western blot images corresponding to Fig. 6a.

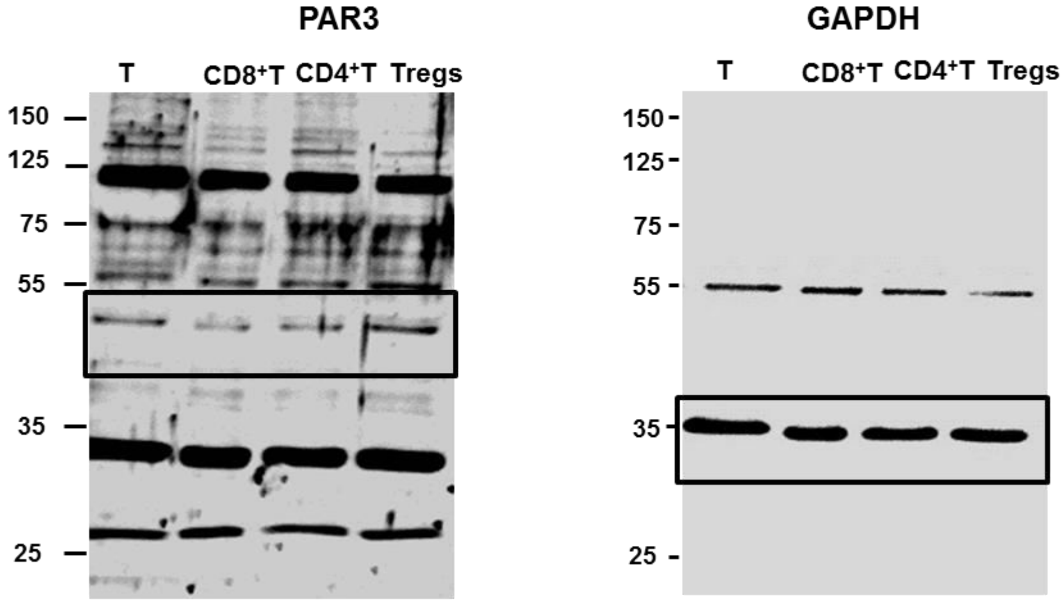

Supplementary Fig. 25: Full western blot images corresponding to Fig. 6b.

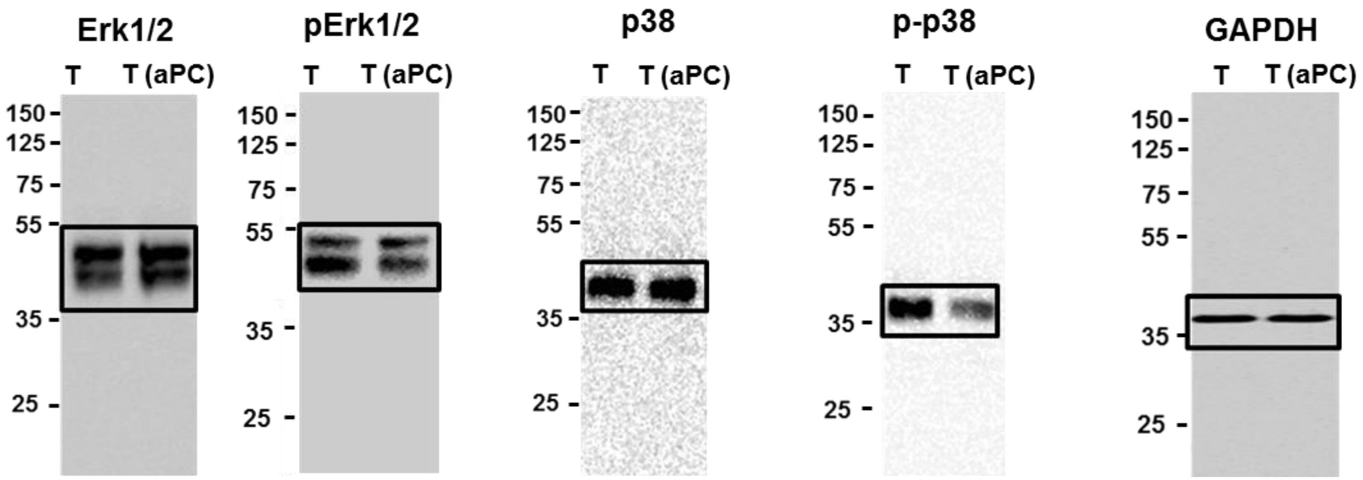

Supplementary Fig. 26: Full western blot images corresponding to Fig. 7c.

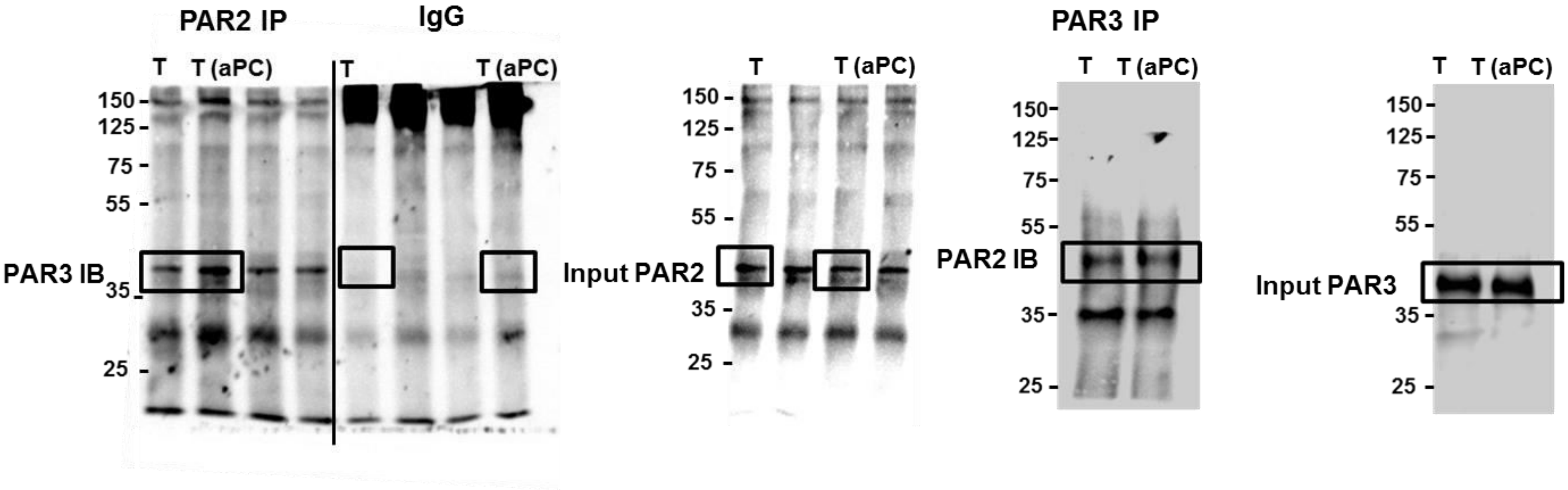

Supplementary Fig. 27: Full western blot images corresponding to Fig. 7d.

Supplementary Table 1

Antibody	Antibody Target	Clone	Host	Dilution	Source
H-2D^b-FITC	mouse MHC class I, H-2D^b	28-14-8	Mouse	1:400	eBioscience
H-2D^b-eFluor450	mouse MHC class I, H-2D^b	28-14-8	Mouse	1:400	eBioscience
H-2K^b-FITC	mouse MHC class I, H-2Kd and H-2Dd	34-1-2S	Mouse	1:400	eBioscience
CD4-FITC	human CD4	OKT4	Mouse	1:400	eBioscience
CD4-APC	mouse CD4	RM4-5	Rat	1:1000	Biolegend
FOXP3-PE	mouse FOXP3	FJK-16S	Rat	1:400	eBioscience
FOXP3-PE-Cy7	human FOXP3	PCH101	Rat	1:400	eBioscience
FOXP3-AlexaFluor647	human, mouse FOXP3	150D	Mouse	1:400	BioLegend
T-bet-PE	mouse, human T-bet	4B10	Mouse	1:200	eBioscience
TNF-α-PE	mouse TNF-α	MP6-XT22	Rat	1:200	eBioscience
IFN-γ-PE	mouse IFN-γ	XMG1.2	Rat	1:2000	eBioscience
IL-10-PE	mouse IL-10	JES5-16E3	Rat	1:200	BioLegend
IL-17A-PE	mouse IL-17A	TC11-18H10	Rat	1:100	BD
Ror-γt-PE	mouse Ror-γt	AFKJS-9	Rat	1:400	eBioscience
T-bet-PerCp-Cy5.5	mouse, human T-bet	4B10	Mouse	1:1000	eBioscience
TNF-α-PE	human TNF-α	MAb11	Mouse	1:200	BioLegend
IFN-γ-PE	human IFN-γ	4S.B3	Mouse	1:1000	BioLegend
IL-10-PE	human IL-10	JES3-19F1	Rat	1:200	BioLegend
IL-17A-PE	human IL-17A	BL168	Mouse	1:200	BioLegend
Ror-γt-PE	human, mouse Ror-γt	AFKJS-9	Rat	1:400	eBioscience
FOXP3	Full-length FOXP3 protein	150D	Mouse	1:1000	BioLegend
PAR1	human, mouse PAR1	ATAP2	Mouse	1:200	Santa Cruz
PAR2	human, mouse PAR2	SAM11	Mouse	1:200	Santa Cruz
PAR3	human, mouse PAR3	H103	Rabbit	1:200	Santa Cruz
PAR4	human, mouse PAR4	H120	Rabbit	1:200	Santa Cruz
EPCR	human, mouse EPCR	RCR-252	Rat	1:500	Sigma-Aldrich
Erk1/2	human, mouse	Polyclonal	Rabbit	1:1000	Cell Signaling
Phospho-Erk1/2	Thr202/Tyr204 human, mouse	Polyclonal	Rabbit	1:1000	Cell Signaling
p38	human, mouse	Polyclonal	Rabbit	1:1000	Cell Signaling
Phospho-p38	Thr180/Tyr182	Polyclonal	Rabbit	1:1000	Cell Signaling
CD25-APC	human CD25	BC96		1:400	BioLegend
CD3	epsilon-subunit within the human CD3 complex	OKT3	Mouse	1μg/ml	eBioscience
CD28	human CD28 molecule	CD28.2	Mouse	0.5μg/ml	eBioscience
GAPDH	Human, Mouse	Polyclonal	Rabbit	1:20000	Sigma-Aldrich
Anti-mouse IgG	Mouse IgG		Horse	1:2000	Cell Signaling
Anti-rabbit IgG	Rabbit IgG		Goat	1:2000	Cell Signaling
